# Non-invasive fetal sex diagnosis in plasma of early weeks pregnants using droplet digital PCR

**DOI:** 10.1186/s10020-018-0016-7

**Published:** 2018-04-05

**Authors:** Elisabetta D’Aversa, Giulia Breveglieri, Patrizia Pellegatti, Giovanni Guerra, Roberto Gambari, Monica Borgatti

**Affiliations:** 1 Department of Life Sciences and Biotechnology, Biochemistry and Molecular Biology Section, University of Ferrara, Via Fossato di Mortara 74, 44121 Ferrara, Italy; 2 Operative Unit of Laboratory Analysis, University Hospital S. Anna, Via A. Moro, 8, 44100 Ferrara, Italy; 3 Biotechnology Center, University of Ferrara, Via Fossato di Mortara 64, 44121 Ferrara, Italy

**Keywords:** Droplet digital PCR, Y-chromosome, Non-invasive prenatal diagnosis, X-linked disorders

## Abstract

**Background:**

Fetal sex determination is useful for families at risk of X-linked disorders, such as Duchenne muscular dystrophy, adrenal hypoplasia, hemophilia. At first, this could be obtained through invasive procedures such as amniocentesis and chorionic villus sampling, having a 1% risk of miscarriage. Since the discovery of cell-free fetal DNA (cffDNA) in maternal plasma, noninvasive prenatal testing permits the early diagnosis of fetal sex through analysis of cffDNA. However, the low amount of cffDNA relative to circulating maternal DNA requires highly sensitive molecular techniques in order to perform noninvasive prenatal diagnosis. In this context we employed droplet digital PCR (ddPCR) in order to evaluate the earliest possible fetal sex determination from circulating DNA extracted from plasma of pregnant women at different gestational ages.

**Methods:**

We identified the fetal sex on cffDNA extracted from 29 maternal plasma samples at early gestational ages, several of them not suitable for qPCR determination, using ddPCR designed for SRY gene target.

**Results:**

All maternal plasma samples were determined correctly for SRY gene target using ddPCR even at very early gestational age (prior to 7 weeks).

**Conclusions:**

The ddPCR is a robust, efficient and reliable technology for the earliest possible fetal sex determination from maternal plasma.

## Background

Currently more than 100 X-linked inherited human disorders have been identified, such as hemophilia, adrenal hypoplasia, muscular dystrophy (Becker, Duchenne and Emery-Dreifuss types), taking a relevant place in the genetic counseling (Germain, 2006). In particular, determination of fetal sex is especially useful in congenital adrenal hyperplasia, permitting the target therapy to female fetuses (New et al., 2014; Sillence et al., 2015)*.* While the fetal sex diagnosis could be obtained using invasive procedures such as amniocentesis and chorionic villus sampling, these procedures are associated with a 1% risk of miscarriage (Sillence et al., 2015).

Non-invasive prenatal diagnosis (NIPD) started after the discovery by Lo et al. (Lo et al., 1997) of circulating cell-freefetal DNA (cffDNA) in maternal plasma. NIPD is based on fetal DNA analysis starting from a simple peripheral blood sampling without disrupting or endangering the health of the unborn child and the pregnant woman, thus eliminating the risks associated with conventional invasive techniques withdrawal.

The Y chromosome was the first marker developed for the detection of circulating cffDNA in maternal blood (Lo et al., 1997). Lo et al. (Lo et al., 1997), through a simple PCR and the use of specific probes for the DYS14 gene, located in single copy on the Y chromosome, demonstrated the presence of fetal DNA in plasma samples of pregnant women bearing male fetuses.

Between 3 and 13% of the total circulating free DNA in the plasma of pregnant women is thought to be fetal DNA (Drury et al., 2016). However, fetal DNA is present in maternal blood at very low concentrations increasing during the progression of pregnancy (Zhou et al., 2015). The proportion of circulating cffDNA grows by 0.1% every seven days between the 10th and 21st week of gestation, then increases faster after the 21st week, reaching almost 1% increment every week (Drury et al., 2016; Zhou et al., 2015). In addition, the amount of circulating fetal DNA depends, besides the gestation period, on other factors, such as maternal diseases and body weight (Zhou et al., 2015; Vora et al., 2012), aneuploidies (Zhou et al., 2015) and twin pregnancies (Attilakos et al., 2011).

Anyway, the very low amount of circulating cell-free DNA in maternal plasma is a very crucial issue and specific and optimized techniques for cffDNA purification from maternal plasma, and very sensitive detection approaches are required.

The most commonly used technology for detecting male fetus-specific DNA in maternal plasma is represented by quantitative real-time PCR (qPCR) amplifying the single copy SRY (sex-determining region Y) gene located on chromosome Y as target gene (Lo et al., 1998), the single-copy sequence DYS14 (Lo et al., 1990) and the multicopy DAZ gene (Stanghellini et al., 2006)*.* However, during early gestation, it is quite difficult to detect very low amounts of cffDNA (Lo et al., 1998; Lo et al., 1990; Stanghellini et al., 2006; Birch et al., 2005)*.*

Devaney et al. (Devaney et al., 2011) reported a systematic review including a PubMed based meta-analysis (January 1, 1997-April 17, 2011), identifying 146 publications used to determine the clinical validity of non-invasive prenatal sex determination using cffDNA in maternal blood and urine based on PCR or qPCR. Despite the expected variability among the considered studies, the overall sensitivity (95.4%) and specificity (98.6%) of the employed technologies were high but when the analytical tests were performed prior to 7 weeks of gestation using blood, they were found to be unreliable (Devaney et al., 2011). Moreover results based on the use of urine samples were demonstrated to be inaccurate (Devaney et al., 2011).

In a recent paper, Breveglieri et al. (Breveglieri et al., 2016) demonstrated that cffDNA, obtained at early gestational ages and not detectable by conventional qPCR for SRY gene target, can be identified with high accuracy and reliability using SPR-based biosensors. In fact samples obtained from maternal plasma at early gestational ages, not detectable by qPCR, were able to generate a positive SPR signal, based on preamplification step of PCR products injected onto Biacore™ sensor chip flow cells, permitting the identification of fetal sex with high accuracy after the 7th gestational week.

In order to develop and validate novel molecular technologies suitable for the earliest possible fetal sex diagnosis, in this report we investigate the possibility to apply the new droplet digital PCR (ddPCR) technology for analysis of cffDNA. The ddPCR is based on water-oil emulsion droplet technology permitting the precise quantification of rare target nucleic acids in the sample (Pinheiro et al., 2012). Droplets are formed in a water-oil emulsion to form the partitions that separate the template DNA molecules. The massive sample partitioning is a key aspect of the ddPCR technique reducing costs, preserving precious samples and detecting rare DNA target copies with high sensitivity (Hindson et al., 2011). In order to identify the male fetal sex, we analyzed, using ddPCR designed for SRY target gene, cffDNAs obtained from 29 maternal plasma samples at the earlier gestational ages available (4.5-12 weeks), not suitable for analysis based on conventional qPCR.

## Methods

### Sample collection

Blood samples from pregnant women were collected by using test tubes containing EDTA anticoagulant after approval by the Ethical Committee of University Hospital S. Anna, Ferrara (Italy). In all cases informed consent was obtained and the experiments were conducted in agreement with the Declaration of Helsinki. A progressive number was assigned to each specimen to ensure the anonymity of the donor.

### Plasma preparation

Plasma was prepared within 3 h from blood collection, according to the protocol previously described in literature (Breveglieri et al., 2016). Briefly, after mixing tubes in a rotator for 5-10 min, samples were centrifuged at 1200×g for 10 min at 4 °C without brake. Plasma was then carefully collected and centrifuged again at 2400×g for 20 min at 4 °C in order to completely remove platelets and precipitates. The resulting supernatant was collected and stored at -80 °C into single-use aliquots.

### Extraction of circulating cell-free DNA

DNA was extracted from 2 mL of maternal plasma, not thawed more than once, by using the QIAamp^®^ DSP Virus Spin Kit (Qiagen, Hilden, Germany), according to the manufacturer’s instructions. DNA elution was performed in 60 μL of AVE buffer.

### qPCR

Circulating DNA extracted from maternal plasma was analyzed using real-time PCR amplification assays for the β-globin gene (forward: 5’-GCAAAGGTGCCCTTGAGGT-3′; reverse: 5’-CAAGAAAGTGCTCGGTGCCT-3′; probe: 5’-FAM/TAGTGATGG/ZEN/CCTGGCTCACCTGG AC/3IABkFQ-3′), and SRY gene (forward: 5’-CCCCCTAGTACCCTGACAATGTATT-3′; reverse: 5’-TGGCGATTAAGTCAAATTCGC-3′; probe: 5’FAM/AGCAGTAGA/ZEN/GCAGTCAGGGAGGCAGA/3IABkFQ-3′) as previously reported (Breveglieri et al., 2016) in order to quantify total and fetal (in case of male fetus) DNA, respectively. Each reaction contained TaqMan^®^ Universal PCR Master Mix (Life Technologies, Carlsbad, CA, USA), in a final volume of 15 μL and was performed in duplicate. The reactions were carried out on a StepOne ^TM^ Real-Time PCR System (Applied Biosystems, Life Technologies), by using the StepOne Software, v2.3 (Applied Biosystems, Life Technologies) and the following amplification program: 2 min at 50 °C; 10 min at 95 °C; 50 amplification cycles comprising a denaturation step at 95 °C for 15 s and an annealing-elongation step at 60 °C for 1 min.

### ddPCR

DNA was detected and quantified using a QX200™ Droplet Digital™ PCR system (Bio-Rad Laboratories, Hercules, CA, USA). Briefly, 11 μL of 2x ddPCR Supermix for Probes (Bio-Rad Laboratories), 1 μL of 20× EIF2C1 Bio-rad assay (ID: dHsaCp2500349, HEX labeled), 1 μL of 62.5× SRY assay (the same reported for qPCR) (Breveglieri et al., 2016) were mixed with 8 or 9 μL of DNA template, according to gestation weeks, in a reaction volume of 22 μL.

To generate the droplets, 20 μL of ddPCR reaction and 70 μL of Droplet Generation Oil for Probes (Bio-Rad Laboratories) were inserted in an eight-well cartridge using a Automated Droplet Generator (Bio-Rad Laboratories) according to manufacturer instructions. Then, 40 μL of the generated droplet emulsion was transferred to a new 96-well PCR plate (Eppendorf, Hamburg, Germany) and amplified in the GeneAmp^®^ PCR System 9700 (Applied Biosystems, Life Technologies). Amplification conditions started with 10 min of activation of DNA polymerase at 95 °C, followed by 45 cycles of a two-step thermal profile of 30 s at 94 °C for denaturation, and 1 min at 60 °C for annealing and extension. A final hold of 10 min at 98 °C was used for the enzyme inactivation. No optimization of ddPCR was necessary with respect to qPCR annealing or probe concentration.

After thermal cycling, plates were transferred to a QX200^TM^ Droplet Reader (Bio-Rad Laboratories). The software provided with the ddPCR system (QuantaSoft 1.3.2.0; Bio-Rad Laboratories) was used for data acquisition to calculate the absolute concentration of target DNA in copies/μL of reaction using Poisson distribution analyses (Pinheiro et al., 2012; Hindson et al., 2011).

## Results

### Experimental strategy

**Fig. 1:**
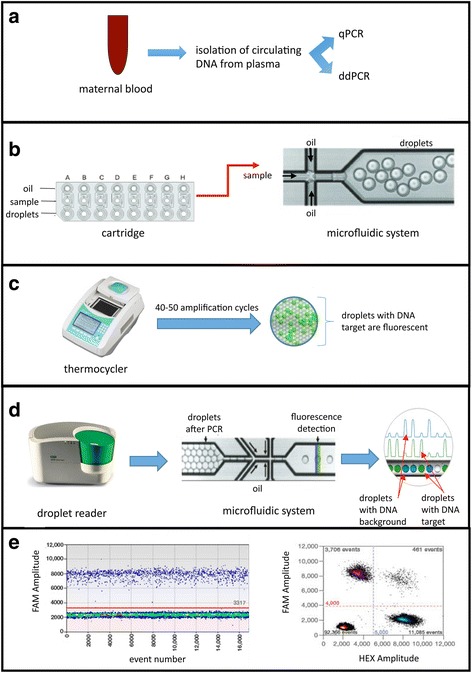
Experimental strategy. **a** Isolation of circulating DNA (maternal and fetal) from plasma obtained from pregant women at different gestational ages and analysis of fetal sex by qPCR and ddPCR. **b** An automated system blends oil samples into a cartridge and generates droplets of nanolitre size by a microfluidic system and vacuum generation. **c** The emulsion is transferred to the thermocycler for the amplification. The presence of the target template in the droplets will be detected by the fluorescence development. **d** Detection of fluorescence using the Droplet Reader. The system, after the injection of a reader oil, separates and aligns each droplet, which is analyzed by two lasers to detect fluorescence. **e** Analysis of the data represented in graphs as the fluorescence (FAM or HEX) relative to each droplet (Event Number)

ddPCR is a recent technology known for its high sensitivity (Pekin et al., 2011). The great sensitivity of the technique is due to the extreme dilution, and hence subdivision of the droplet sample consisting of an oil-based emulsion generated by the QX200™ AutoDG™ Droplet™ Digital™ PCR System. If the dilution is correct within each of the aliquots, a single amplification reaction will be performed, increasing the method sensitivity (Corbisier et al., 2015). For this reason, ddPCR was used in this study as an alternative approach for the identification of fetal sex at very early gestation weeks, where conventional qPCR was unable to discriminate male from female fetuses. In particular, cffDNAs were extracted from 29 maternal plasma samples at very early gestational ages (4.5-12 weeks) using the QIAamp^®^ DSP Virus Spin Kit (Qiagen) and analyzed by qPCR and ddPCR for the sex determination (Fig. 1).

### Determination of fetal sex from maternal plasma by qPCR

The most common method employed to determine fetal sex is qPCR, due to its sensitivity, specificity and easiness of application (Lo et al., 1998). In our case, qPCR was used as the first approach to fetal sex determination of 29 plasma samples obtained from pregnant women at early gestational ages. A specific test for the SRY gene was designed according to literature (Lo et al., 1998). This assay was used to amplify male DNA, as the SRY gene is located only on the Y chromosome. The human β-globin gene was used as reference gene for the quantification both fetal and maternal DNA, confirming the presence of extracted circulating DNA. For these specimens, whose gestation weeks are between 12 and 4.5, the fetal sex diagnosis was unreliable for all, with the exception of 8 samples on 29 (Table 1).

**Table 1:** Fetal sex determination of circulating cell-free DNAs by qPCR and ddPCR. The actual fetal sex and the diagnostic outcome after fetal sex determination by qPCR and ddPCR were determined for circulating cell-free DNAs obtained from 29 pregnant women at different gestational ages. The samples 1A and 1B were obtained from the same pregnat woman at two different gestational weeks. *M* male, *F* female. *N.D.* not determinable

# sample	Gestational week	Result at birth	Result by qPCR	Result by ddPCR
1A	12	M	N.D.	M
2	12	M	N.D.	M
3	11	M	N.D.	M
4	10	M	N.D.	M
5	10	F	F	F
6	10	M	M	M
7	10	M	N.D.	M
8	9.5	M	N.D.	M
9	9	F	F	F
10	8	F	F	F
11	8	M	N.D.	M
12	8	M	N.D.	M
13	8	M	N.D.	M
14	8	M	N.D.	M
15	8	M	N.D.	M
16	8	F	F	F
17	8	M	M	M
18	7	M	N.D.	M
19	7	M	N.D.	M
1B	7	M	N.D.	M
20	7	M	N.D.	M
21	7	M	N.D.	M
22	7	M	N.D	M
23	7	F	F	F
24	7	M	N.D.	M
25	6	M	N.D.	M
26	6	M	N.D.	M
27	5	M	N.D.	M
28	5	F	F	F
29	4.5	M	N.D.	M

These results confirmed the data reported in literature, pointing out that the qPCR testing is not reliable and accurate for fetal sex diagnosis when performed using blood withdrawn at early gestational period, in particular prior to 7 weeks of gestation (Devaney et al., 2011; Breveglieri et al., 2016).

The difficulty of qPCR-based methods to identify male fetal sex at early gestational stages is due to (a) absence of double amplification curves for the SRY gene, creating false negative; (b) generation of only one amplification curve caused by SRY amplification or contamination, preventing a robust and correct diagnosis; (c) presence of double amplification curves not superimposed with ΔCt > 0.5 (Yuan et al., 2006), generating an unreliable quantification. Anyway, in all the cases, the described problems are due to the insufficient amount of circulating fetal DNA present in maternal plasma in early gestational weeks (12 weeks or less) (Zhou et al., 2015). In conclusion, despite the sensitivity and specificity of the qPCR method, this technique has limitations for robust and reliable diagnosis of fetal sex when a reduced amount of template is available. Therefore, for earlier prenatal diagnosis of fetal sex, we hypothesized that more sensitive and efficient techniques, such as ddPCR, could be a valid and alternative approach.

### Set-up of ddPCR conditions

In order to identify the fetal and total circulating DNA, two amplification assays were used in ddPCR, both consisting of two primers (forward and reverse) and a hydrolysis probe each with a fluorophore. The first assay, labeled with the FAM fluorophore, is specific for the SRY gene, and is exactly the same assay previously used in qPCR (Breveglieri et al., 2016), applied to ddPCR. Fetal sex was considered male or female depending on whether or not positive FAM fluorescence events, corresponding to the SRY gene, were detected.

The second assay, labeled with the HEX fluorophore, specific for the EIF2C1 gene, is located on the chromosome 1 coding for an argonaute protein and considered as reference gene in order to confirm the actual presence of circulating DNA in the absence of positive events for SRY. Since the assays are marked with two different fluorophores, both amplifications were performed in the same reaction, with the advantage of increasing the precision of the analysis and using half of the starting material.

**Fig. 2:**
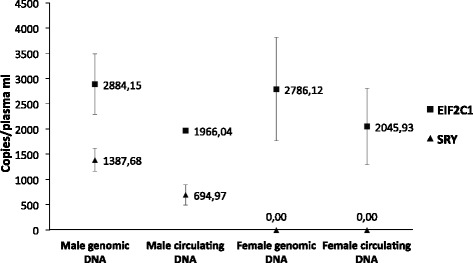
ddPCR analysis on male and female circulating and genomic DNA samples in order to set-up the SRY assay. The concentration average (from three different subjects for each category) of SRY (dots) and EIF2C1 (squares) was expressed in copies/μL of reaction, obtained through QuantaSoft software, based on Poisson’s statistics

To test the correct performance of the two assays and to identify the optimum amplification conditions, a preliminary set-up experiment was performed (Fig. 2). In this experiment, male/female genomic DNAs and circulating DNAs from male or female adults (obtained from three different subjects for each category) were analyzed. Male genomic DNA was considered as positive control: it is expected to have positive events both for the SRY gene and the EIF2C1 gene in a ratio of 1:2, since the SRY gene is present in a single copy relative to the duplicate EIF2C1 gene. Female genomic DNA was considered the negative control: it is expected to have no positive events for the SRY gene, since female subjects do not possess this gene. Finally, the male and female circulating DNAs were used as a further positive and negative control respectively, since they are more similar in terms of fragmentation to cffDNA extracted from maternal plasma than genomic DNA. Figure 2 shows the absolute quantification obtained using the described four samples and the SRY target and EIF2C1 reference genes.

The SRY gene for male genomic DNA was calculated to be 1387.68 copies/μL of plasma, whereas the EIF2C1 content was 2884.15 copies/μL of plasma with a ratio 1:2, due to the fact that the EIF2C1 gene is present in duplicate, while the SRY gene, being present only on the male sexual Y chromosome, is present as a single copy. This ratio is not maintained in the circulating DNA of an adult male. In fact, EIF2C1 quantification of 1966.04 copies/μL of plasma and 694.97 copies/μL of plasma for SRY were observed. The apparent inability to maintain the 1:2 ratio is caused by the quality of the sample, which, being circulating DNA, is extremely fragmented, with small size and easy degradation (Drury et al., 2016). On the other hand, in the case of female genomic and circulating DNAs, SRY is not present and its concentration is equal to 0, demonstrating that sex can be discriminated in the used experimental conditions. Furthermore, the EIF2C1 quantification is similar to the corresponding male samples, confirming the different nature and variability of genomic and circulating DNAs. In any case, we like to point out that, as previously discussed, for the diagnostic identification of sex, it is not essential to obtain the same amount of circulating DNA present in maternal plasma, but only the confirmation of its presence or absence is required.

### Determination of fetal sex from maternal plasma by ddPCR

The 29 maternal plasma samples at early gestational stages, previously analyzed by qPCR (Table 1), were quantified for SRY gene target using ddPCR in order to identify the fetal sex.

**Fig. 3:**
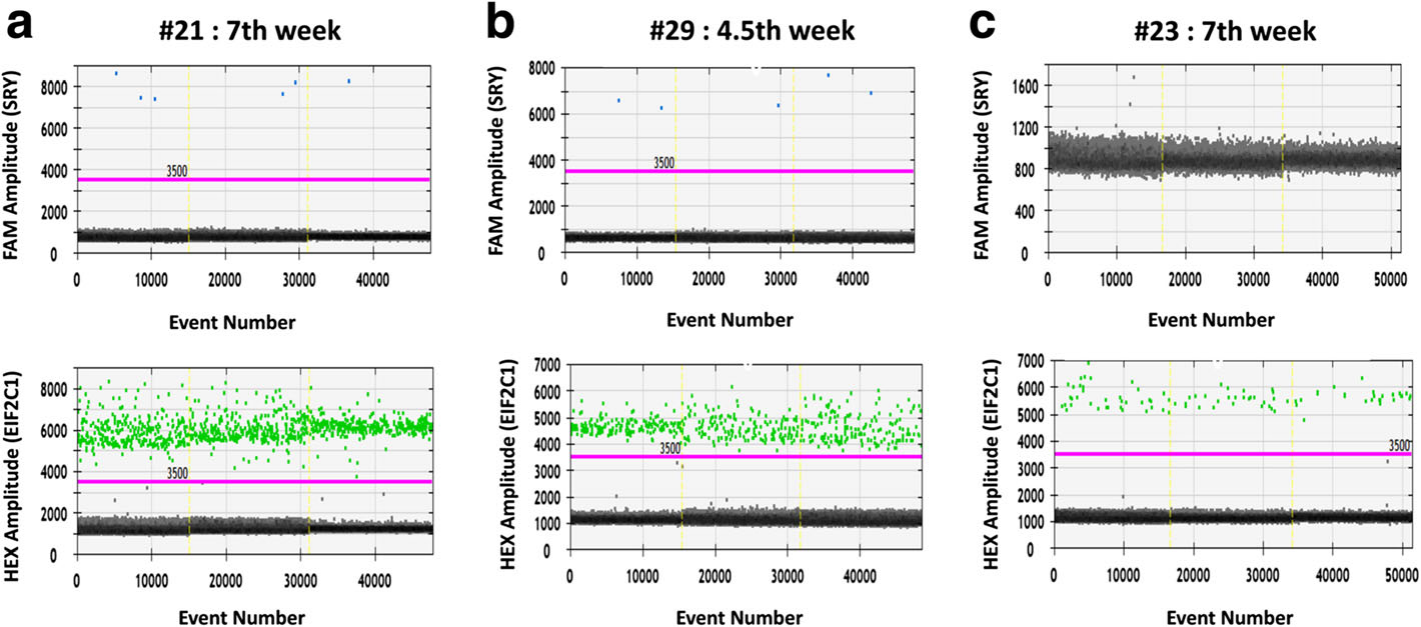
Representative graphs for circulating DNA samples at different gestational ages analyzed by ddPCR. Three circulating DNA samples, extracted from maternal plasma at different gestational weeks and carrying male (**a**, #21: 7th week; #29: **b**, 4,5th week) or female fetus (**c**, #23:7th week), were analyzed in ddPCR for SRY (FAM) and EIF2C1 (HEX) gene targets. For all three samples, the upper graphs correlate the FAM fluorescence intensity, corresponding to the SRY gene (blue dots), to the number of positive events; the lower graphs report the HEX fluorescence intensity, relative to the EIF2C1 reference gene (green dots), to the number of positive events. Black dots indicate negative events (no amplification events). The threshold lines are coloured in fuchsia

Figure 3 shows the representative graphs obtained from maternal plasma samples at the 7th (Fig. 3a) gestational week carrying male or female fetus (Fig. 3a and c respectively) and at the 4.5th week having male fetus (Fig. 3b).

The upper graphs show the events in which the SRY amplification reaction gene occurred correlating the FAM fluorescence intensity with the number of observed events (blue dots); while the lower graphs identify the events in which the EIF2C1 gene was amplified relative to the HEX fluorescence intensity with the number of events (green dots).

In Fig. 3a and b, the limited number of positive events related to the SRY gene observed for samples #21 and #29 (7th and 4.5th gestational week, respectively) are still sufficient to perform a robust and reliable analysis being more than 3. These data are due to the decrease in the concentration of fetal DNA circulating in maternal plasma at decreasing gestational time (Zhou et al., 2015). While, for the sample #23 at the 7th week carrying a female fetus (Fig. 3c), no positive events of SRY amplification have been observed reporting only negative events (black dots). In the lower charts, the positive events relative to the HEX fluorescence intensity correlated to the EIF2C1 gene amplification, were detected for all three samples and were significantly than the SRY gene quantification confirming the template presence in the reactions.

Table 1 (fourth column) summarizes the results of fetal sex determination obtained by ddPCR for the 29 maternal plots analyzed. For all the samples analyzed in ddPCR, it was possible to determine and confirm the birth sex also for maternal plasma with male fetus at early gestation weeks not validated in qPCR (Table 1, third column).

## Discussion

In recent years, the cffDNA in maternal plasma has been extensively investigated for detecting prenatal disorders and pregnancy monitoring (Tounta et al., 2011; Galbiati et al., 2005; Edlow & Bianchi, 2012; Pescia et al., 2017). In particular, the fetal sex determination is fundamental in the NIPD field for sex-related diseases, as it allows predicting the pathological phenotype for the unborn, thus avoiding invasive diagnostic procedures for pregnant women with no risk to the fetus. In the specific case, for recessive X-linked diseases, the possibility of pathological phenotype for female fetuses is excluded, while for males the risk persists. The most commonly used technology for detecting male fetus-specific DNA in maternal plasma is represented by qPCR (Lo et al., 1998; Lo et al., 1990; Stanghellini et al., 2006), but analysis performed prior to 7 weeks of gestation using cffDNA from maternal plasma were unreliable (Devaney et al., 2011). Therefore, the development of more sensitive, specific and earlier technologies are investigated for the determination of fetal sex from cffDNA in maternal plasma, such digital PCR.

The technique allows to amplify a single DNA template from minimally diluted samples, therefore generating amplicons that are exclusively derived from one template and can be detected with different fluorophores or sequencing to discriminate different alleles (Pohl & Shih, 2004; Hudecova, 2015). Different technologies for digital PCR have been developed: microfluidic integrated circuits for partitioning samples (Spurgeon et al., 2008); bead-based emulsion PCR technology (BEAMing: beads, emulsion, amplification, magnetics) (Diehl et al., 2006); droplet-based partitioning, which partitions a sample into 20,000 droplets and digitally counts nucleic acid targets (Pinheiro et al., 2012; Hindson et al., 2011); picoliter-scale droplet technology, which generates up to 10 million picoliter-sized droplets per lane (Pekin et al., 2011).

For example, Karakas et al. (2015) reported a digital PCR technology called BEAMing for detecting male cffDNA from the maternal plasma at 2 to 6 weeks following embryo transfer (i.e., 4 to 8 weeks pregnancies) by targeting Y chromosome-specific sequences of the amelogenin gene (AMELY).

Lun et al. (2008) used microfluidic digital PCR to quantify the male fetal DNA in first-, second- and third-trimester maternal plasma amplifying two 87-bp amplicons of the ZFX (zinc finger protein, X-linked) and ZFY (zinc finger protein, Y-linked) loci.

In this paper we applied the droplet digital PCR (ddPCR, Bio-Rad Laboratoires) to quantify SRY gene target from cffDNA at the earliest gestational ages. This system partitions nucleic acid samples in thousands of nanoliter-sized droplets, and uses lower samples and reagent volumes than other commercially available digital PCR systems (Pinheiro et al., 2012; Hindson et al., 2011).

For the first time, we have demonstrated that ddPCR technology can be used for NIPD of Y chromosome at early gestational ages (prior to 7 weeks). In particular, all maternal plasma samples were determined correctly for SRY gene presence using ddPCR even at earliest gestational age (4.5 weeks), achieving 100% accuracy. To our knowledge, previous to this work, the earliest reliable cffDNA detection time point was 7 weeks in pregnancy (Devaney et al., 2011) using qPCR, but no studies are reported for ddPCR at so early week pregnancy.

## Conclusions

In conclusion the ddPCR is a robust, efficient and reliable technology for the earliest possible fetal sex determination from maternal plasma.

The efficiency of ddPCR allows to extend its applicability to other very important field of biomedicine, such as liquid biopsies in tumor patients (where the presence of tumor-free circulating DNA content is similar to fetal circulating DNA) (Wu et al., 2017).

## Abbreviations

cffDNA: cell-free fetal DNA
ddPCR: droplet digital PCR
NIPD: non-invasive prenatal diagnosis
qPCR: quantitative real-time PCR
SRY: sex-determining region Y
